# Patterns of presentation of adults with hearing impairment in a peri-urban community in South Africa: a qualitative study

**DOI:** 10.1186/s12913-023-10025-5

**Published:** 2023-09-21

**Authors:** Thobekile Kutloano Mtimkulu, Katijah Khoza-Shangase

**Affiliations:** Department of Audiology, School of Human and Community Development, University of the Witwatersrand, Johannesburg, South Africa

**Keywords:** Behavior patterns, Help-seeking, Hearing impairment, South Africa, Context, Culture

## Abstract

**Background:**

There is a wealth of research, globally, on the help-seeking behavior patterns of individuals with a variety of chronic medical conditions. Contextually relevant findings have been reported describing the influence of a disease and/or disorder together with the related personal, social, environmental, and region-specific factors. However, research related to help-seeking behavior patterns in hearing impairment is mostly found in high income countries (HICs) and tells a one-sided story indicating a knowledge gap in other contexts. As part of a bigger study titled “In pursuit of preventive audiology: Help-seeking behavior patterns of adults with hearing impairment in a peri-urban community in South Africa”, the main aim of this study was to describe the patterns of presentation of symptoms in adults with hearing impairment from a peri-urban community in South Africa.

**Methods:**

Through a non-probability purposive sampling method, 23 adults with hearing impairment participated in semi-structured interviews using an interview guide from April – June 2021. Thematic analysis through a deductive analysis approach was adopted for data analysis.

**Results:**

Participants’ patterns of presentation indicated a belief in a Western bio-medical causation to explain their hearing impairment. Help-seekers sought help mostly from healthcare workers at all levels of healthcare in the public (primary, secondary, and tertiary) and private healthcare sectors, followed by a few participants consulting their social networks. Only one participant used a traditional healthcare provider to resolve his hearing difficulties.

**Conclusions:**

In a diverse country like South Africa, with a majority African population, the influence of cultural practices and beliefs proved to have a minimal influence on participants’ help-seeking behavior for their ear and hearing challenges. However, an interplay of factors related to one’s context at the time played a significant role in the patterns of presentation to the ear and hearing clinic. There is therefore a need to understand the perspectives of adults with hearing impairment from their environments to facilitate more contextual relevancy in healthcare provision within the preventive audiology field. Hearing health policymakers should also endeavor to consider the realities of health-seeking in each context and environment.

**Supplementary Information:**

The online version contains supplementary material available at 10.1186/s12913-023-10025-5.

## Background

Hearing impairment is a silent and an invisible disability affecting one in five people globally [1, 2]. The greatest burden of this chronic disease is reportedly carried by low-and-middle-income countries (LMICs) with the World Health Organization (WHO) [3] estimating nearly 80% of those living with a disabling hearing impairment residing in these countries. In South Africa, despite being over 10 years, the latest published results of the population census of 2011 found hearing impairment to be the third highest disability [4].

Adults living with a disabling hearing impairment experience marked social, emotional and communication difficulties [5]. This can lead to stigma, frustration, social isolation to name a few negative sequelae of hearing impairment [6, 7]. The impact of this disorder on their quality of life together with the continuing signs and symptoms results in a sequence of events that leads to decisions having to be made to resolve those difficulties [8, 9]. As a result, in seeking help, individuals may present their symptoms to their social networks (formal and informal) to find solutions to their bodily dysfunction and activity limitations [10, 11].

Contingent on the individual, the disease/disorder and the context, the help sought can result in a pattern especially in countries with diverse socio-cultural beliefs about illnesses, multiple health services and challenges in access and availability of healthcare [12, 13]. This description can be labelled as a pattern of presentation among individuals, communities, and population groups in specific environments. South Africa is one such country that can be described as culturally and linguistically diverse with challenges in healthcare that include a quadruple burden of disease [14, 15]. A study [12] comparing help-seeking pathways to healthcare in HICs and LMICs found a different pattern of presentation between the two country categories. Although focused on mental illness, this study indicated that general practitioners and mental health professionals were central in the pattern of presentation for help-seekers in developed contexts whereas traditional healers were predominant in LMICs [12].

Research on hearing help-seeking patterns, though limited, has displayed a one-sided view of this phenomenon. Much of the available evidence emanates from HICs contexts with individualistic societies having equitable patterns of living and access to healthcare [6, 8, 16–18]. In these studies, individuals with hearing difficulties presented their symptoms to general practitioners, audiologists and Ear, Nose and Throat (ENT) Specialists [6, 8, 19]. The direct availability and access to the medical professionals because of the structure of the healthcare systems was also a contributing factor to this pattern of presentation [6]. Nevertheless, none of the studies investigated other possible health providers that individuals might have contacted before seeking help from these formal networks.

Evidence from South African research on non-auditory chronic diseases such as tuberculosis, hypertension, and cancer describe individuals presenting to a variety of medical providers such as public healthcare clinics (PHC), hospitals, private or public healthcare providers [13, 20, 21]. Furthermore, countries with multi-cultural and multi-lingual contexts like Nigeria, India and Uganda reported religious leaders, herbal and alternative providers as the first pattern of presentation for help-seekers, indicating a preference towards non-medical health providers [9, 22, 23]. This contrasts with a study conducted in Pakistan where the majority of participants presented to medical doctors [24]. However, before even reaching the medical doctor, female participants initially sought help from their partners because of the traditional and patriarchal system in that country. These findings point to a complex process where decisions towards seeking help for ill health are not homogenous for all contexts [10, 25], and thus highlight the importance of establishing such patterns for each context.

Although the medical conditions mentioned present differently to hearing impairment, evidence from the reviewed studies reveal that the pattern of presentation of symptoms is not linear but rather influenced by other factors in the social context [26]. People emerge from cultural contexts, and this has an impact on decision-making [27, 28]. For this reason, as evidence from other non-auditory conditions demonstrates, there is not a single pattern that defines the pathway of presentation, and awareness of this pattern for a country is important for appropriate and contextually responsive preventive initiatives including health education and promotion programs. Therefore, this study aimed to describe the pattern of presentation of symptoms of hearing impairment in adults in a peri-urban community in South Africa, in relation to one’s cultural practices and beliefs. The deliberate focus on cultural practices and beliefs was due to the reports that over 80% of South Africans consult traditional healers, particularly for primary healthcare requirements [29]. As part of a bigger study titled “In pursuit of preventive audiology: Help-seeking behavior patterns of adults with hearing impairment in a peri-urban community in South Africa”, the study forms part of efforts towards preventive audiology initiatives within the South African context.

## Methods

### Study design

The aim of this study was to investigate the patterns of presentation of symptoms of adults with hearing impairment in relation to their cultural practices and beliefs. In a multi-cultural and diverse context where ill-health can be resolved in a variety of ways, the researchers sought to understand the influence of the environment in seeking help for ear and hearing symptoms.

Therefore, this study adopted a qualitative descriptive research design to access the thoughts and feelings of research participants through the sharing of their experiences of presentation of ear and hearing symptoms [30]. The descriptive nature of the design provided a comprehensive summary of participants’ practices by staying closer to the data and the actual words used. This led to thicker descriptions and deeper meanings which contributed to the credibility of the data [31–33].

### Study site and sampling

Participants who were scheduled for their first audiology assessment of their hearing impairment were pre-selected using a non-probability sampling method and a set of inclusion and exclusion criteria [34]. The inclusion criteria consisted of individuals from any gender who were over the age of 18 years and presented with a hearing loss regardless of type, degree, or severity. Participants were excluded when they were younger than 18 years of age and were unable to consent and participate in the study due to cognitive and linguistic challenges.

The departmental diary of a public hospital with an audiology department in Potchefstroom, in the North West province was used as a reference for identifying participants. This site was used as the main researcher (TKM) had access to potential participants from the audiology department as an employee of the hospital. On the day of the assessment, potential participants were approached, provided with the research study information and the option to consent to be part of the study. After accounting for maximum variation in the sampling of participants, 23 individuals were included in the study [32]. The initial aim for the study was to have 30 participants, however the sampling size was influenced by data saturation [35]. Data was deemed as truly saturated for a category when no additional data were being obtained in response to any question. As soon as the researchers found similar responses repeated over and over again, confidence was gained that data saturation had occurred. In addition, because of the qualitative nature of the study, it was important to remember that the purpose of the study was to understand the phenomenon under study through in-depth, detailed information as opposed to making generalizations through a large data sample [32, 33].

### Participant and data collection

Individual semi-structured interviews were conducted, and audio recorded by TKM using an open-ended interview guide (Appendix A) developed according to the aim of the study as well as questionnaires from similar research studies. TKM, as the lead researcher and interviewer, is a practicing speech pathologist and audiologist who worked at the research site during the study but was not involved in the assessment and management of the audiology participants forming part of the current study. KKS is the main supervisor of the study with preventive audiology within the African context as her research niche. Participants were asked to describe, using their own language, the onset of ear and hearing symptoms, the first contact in seeking help, the action and management provided and the reasons for using this contact. Although semi-structured interviews pose a particular demand on participants’ memory and ability to recall, the interviews assisted in producing powerful insights - deeper, richer understanding of the phenomenon - into participants’ experiences of the process of seeking help for their hearing impairment [36]. The interviewer was also able to probe responses from participants’ views on the subject [33, 37]. However, it was important during interviews that the interviewer, who was an employee in the same setting, constantly remind herself of her position as an investigator to minimize bias even when patients reflected on the audiology session. Reflecting with the other researchers and through using a diary to document her experiences helped the lead researcher to limit influencing and being influenced by the participants’ lived experiences. All participants were over the age of 18 years and were able to answer the questions independently or with the assistance of a family member. For most of the participants, interviews were conducted face-to-face at a convenient time before or after their audiology assessment. Telephonic interviews were also conducted for those participants who were missed during the data collection period, but only where the hearing impairment did not pose a barrier to communication. All data collection took place from April until June 2021. This was deemed a reasonable amount of time to reduce the risk of inconsistencies and reliability in the data collected. Due to the COVID-19 pandemic at the time of data collection, all health and safety and infection control precautions were adhered to according to stipulated regulations [38, 39].

A pilot study was also conducted to ensure that the research design, methods and sample were appropriate, for reliability and validity assurance [33]. Additionally, measures of ensuring reliability and validity included careful and consistent data collection procedures, as well as data analysis that included an independent rater who also transcribed and re-coded the data independently, prolonged engagement with the data, as well as member checks, as measures of truthfulness of the data.

### Data analysis

All recorded data were transcribed verbatim, translated into English, and analyzed thematically using a deductive analysis approach [40]. The researcher (TKM) used an Excel spreadsheet to load and manage the data. Once transcribed, multiple readings of the raw data were conducted for the purposes of familiarizing oneself with the content before it could be broken down [41]. In analyzing the data in this way, the participants’ stories were truthfully captured in relation to the research question [42]. After multiple readings, the raw data was then coded for topics, issues, differences, and similarities in the participants’ narratives [30]. This process of multiple readings increased the reliability of the transcribed data. Codes were then collated and condensed into categories [40, 43]. As result of wanting to re-test existing data in a new context, based on previous research investigations, coding was also conducted to correspond with the identified categories in the literature [44]. However, the researcher was aware that new categories could emerge from the data based on the context. A large amount of data was generated from the interviews, which therefore required a process of refining and revising the emerging categories to reduce redundancy [45, 46]. An independent person also transcribed and re-coded the data independently as a measure of truthfulness and to increase the reliability of the data [30, 42]. Rich, thick descriptions from the interviews as transcribed in the results gave additional evidence to the credibility of the data. Any disagreements and differences from the analysis were discussed and resolved. The identified categories formed the basis for the results and discussion section of the paper [41].

Measures of central tendency were also used to analyze the quantitative data from the participants’ socio-demographic information using mode, median and mean measures [33].

### Ethical considerations

Ethical approval was obtained from the University’s Research Ethics Committee (Medical) (protocol number: ME 201,003) and the Department of Health Research, Monitoring and Evaluation Directorate before the study could commence. Ethical considerations that guided the current study were based on the World Medical Associations (WMA) Declaration of Helsinki [47]. Specifically, informed consent was obtained from all participants in their own language. Data were collected in a separate room to ensure confidentiality and privacy. Participants were informed that they could stop the interview at any moment without any negative consequences to them. The health and rights of the participants were also upheld throughout the study.

## Results

The results describe the patterns of presentation of symptoms of adults with hearing impairment in relation to their cultural practices and beliefs (Table 1). With an almost equal gender representation and context demographic profile representative sample, all participants were diagnosed with a hearing impairment ranging from 23,75 dBHL to 91,25 dBHL in the better ear, calculated as a four-frequency pure tone average (4FPTA). The mean of the better ear indicated a moderate hearing impairment [48]. Table 2 provides a more detailed description of each participant from the data analysis.

**Table 1:** Participant Sample Characteristics

Participant Sample Characteristics (N = 23)	
Age, years (M ± SD)	67.8 ± 15.6
Gender %	
Male	52
Female	48
Ethnic group %	
Tswana	57
Afrikaner	39
English	4
Occupational Status %	
Employed	9
Unemployed	91
Consultation %	
Alone	57
Accompanied	43
PTA (0.5 Hz – 4 kHz)	
Better ear (SD)	49.6 dBHL (9.1)
Worse ear (SD)	56.1 dBHL (8)

*Key*: M = mean, SD = standard deviation, PTA = Pure Tone Average, dBHL = decibel hearing level

**Table 2:** Participant Profile

Participant No.	Gender	Age	Ethnicity	Degree of hearing loss*
1	Male	73	English	59 dBHL
2	Female	89	Tswana	42 dBHL
3	Female	53	Tswana	70 dBHL
4	Female	82	Afrikaner	41 dBHL
5	Female	78	Afrikaner	32 dBHL
6	Female	68	Afrikaner	32 dBHL
7	Female	28	Tswana	60 dBHL
8	Male	64	Tswana	27 dBHL
9	Male	68	Afrikaner	21 dBHL
10	Female	78	Tswana	21 dBHL
11	Male	87	Afrikaner	24 dBHL
12	Male	82	Afrikaner	69 dBHL
13	Male	71	Tswana	69 dBHL
14	Male	75	Tswana	54 dBHL
15	Female	48	Tswana	29 dBHL
16	Male	56	Tswana	70 dBHL
17	Male	65	Tswana	27 dBHL
18	Male	40	Tswana	22 dBHL
19	Female	80	Afrikaner	66 dBHL
20	Male	65	Tswana	25 dBHL
21	Female	56	Afrikaner	51 dBHL
22	Male	89	Tswana	86 dBHL
23	Female	64	Afrikaner	36 dBHL

*PTA better ear (dBHL)

Participants’ patterns of presentation, prompted by physical ear and hearing symptoms as well as self-reported hearing difficulties, were between formal and informal networks. As depicted in Table 3 below, the majority of participants (82,6%) reported that they presented themselves to Western medical healthcare professions with only 17,4% seeking help from family, friends, and colleagues.

**Table 3:** Participants’ pattern of presentation

First contact in seeking help	Number of participants	Percentage
Public Healthcare Clinic	6	26.08
Private Doctor	4	17.39
Family and Friends	3	13.04
ENT Specialist	2	8.7
Audiologist	2	8.7
Hearing Centre	2	8.7
Public Hospital Endocrine Specialist	1	4.34
Private Healthcare Worker	1	4.34
Traditional Health Provider	1	4.34
Chemist	1	4.34

These formal networks were mostly the PHC as well as private healthcare doctors, with audiologists and ENT Specialists being the least reported points of help-seeking. The PHC is the first entry point into the public healthcare system hence the higher number of participants presenting themselves there. Participants who sought help at the PHC would likely have been helped by a nurse, with just a basic ear examination (otoscopic examination), as ear and hearing care specialists are commonly found in hospitals, less so in PHCs.

Participant 15 stated:

*“I went to the clinic.’ (Participant 15)*.

Participant 24 reported the following about his journey:

*“I only started seeking help in the previous week (pauses) I was at the clinic:” (Participant 22)*.

Those who did not use public healthcare clinics reported the following:

*“We first went to the doctor.” (Participant 2)*.

Participant 10’s daughter responded in this way:

*“She went to the GP first…then after the GP she then went straight to the specialist doctor.” (Participant 10)*.

The PHC, as the route for seeking help in the public health sector indicated what was available for participants in this context. Similarly, private doctors were first in line for those consulting in the private health sector.

Part of the pattern of presentation also involved participants seeking help from their social support networks. These social support networks nevertheless mostly directed them towards healthcare professionals.

Participant 9 stated:

*“I told the lady that stayed with me…’ (Participant 9)*.

Participant 23, referred to her neighbor who was also her friend as the one whom she first asked for help by stating that:

*“She referred me; they will do it at (hospital name) soon.” (Participant 23)*.

Interestingly, only one participant reported an ‘alternative’ traditional practice as a pattern to resolve his hearing difficulties. This participant, a 58-year-old male, felt strongly about being an African and the associated use of indigenous medicine as a remedy for his symptoms. He reported the following:

*“I found help when I used a traditional remedy from a colleague who was a traditional medicine provider.” (Participant 16)*.

It is important to note that those participants that presented to ear and hearing care specialists did so because of previous knowledge through family and friends who had used those services or had worked with the healthcare workers.

### Pattern of presentation in relation to cultural practices and beliefs

In relation to cultural practices and beliefs, participants’ responses pointed towards a belief in the Western medical model to cure illness. The majority of the participants (82,6%) expressed the value of healthcare professionals over traditional or cultural practices to resolve their hearing complaints. This view was found in participants from different ethnic groups, with different ages and socio-economic position within the sample. Notably, some participants appeared surprised at the question, nevertheless they felt strongly about their beliefs by saying:

*“You go to the doctor. Even with your beliefs, you must go see the doctor, we refer to you because you guys went to school.” (Participant 17)*.

Participant 2’s daughter reported that:

*“No, we went to the doctor because we thought that he would have some knowledge.” “For us, we think that the doctor has the knowledge, or the knowledge would be from the doctor.” (Participant 2).*

Participant 22, explained his medical journey and negative view towards traditional practices by stating the following:

*“I believe that I want to be healed, feel better, cured.” “It is you, the doctors, that will cure me. Traditional healers will only take advantage of your money.” (Participant 22)*.

However, Participant 16 contrasted this view by explaining his choice of seeking help:

*“We are people of the soil, those Africans that believe in African remedies, that is why I used the remedy that was given to me.” (Participant 16)*.

This participant did not hesitate to use this remedy as it was the norm and formed part of his socio-cultural context. No other participant in the sample reported accessing help through faith healers or any other traditional or religious practices.

Participants (13%) that had sought help from informal networks like family and friends, had themselves assumed that they had made first contact with a healthcare professional but in fact they had first sought help from non-healthcare networks. This difference was observed as participants were reviewing their responses during the interviews. Surprisingly, the number of participants (13%) that were influenced by their immediate social support was more than those (4%) from traditional practices. Two participants reflected on their help-seeking by stating that;

*“OK, I can start with that, because I’ve had a hearing problem for quite several years now and I’ve been asking my neighbor because her mother used to be here.” (Participant 23)*.

Neighbors and members of the church were the first to be confided in for help. Evidently, social networks were valued in this context to influence the trajectory of their help-seeking journeys. Participants did not directly present themselves to healthcare professionals but rather non-healthcare networks for support.

However, data analysis also indicated that even though some participants presented to healthcare professionals or their social networks, they nevertheless highlighted and defended their beliefs and therefore appeared to justify the reason for consulting medical professionals.

*“…but I use medical doctors, that is where my faith is and then also prayer (pauses) those two things.” (Participant 8)*.

He also stated:

*“I go with the belief that I am going to pray to God to ask where should I start? Yes, to help me and to help the doctors because if you don’t pray to God to help my doctors, sometimes things won’t work out.” (Participant 8)*.

Participants acknowledged their journeys were influenced by their beliefs. Yet this was only admitted in the interview process as patients were being probed and were comfortable expressing themselves before the researcher. It is possible that the hospital context influenced participants’ responses to be more socially correct and provide what they deemed socially desirable responses for the Western medical context they found themselves in at the time.

## Discussion

The main objective of this study was to describe the pattern of presentation of symptoms in adults with hearing impairment from a peri-urban community in South Africa. Findings from this study reveal a pattern in which adults with hearing impairment in South Africa (82,6%) present themselves to the formal, Western medical healthcare providers for resolving their hearing difficulties. Despite the diverse country context, multiple health services, socio-economic differences, challenges in access and equity in healthcare; an understanding of hearing impairment from a biomedical causation requiring the same solution is strikingly indicated [9, 13, 49]. This is consistent with other studies conducted in South Africa [20, 21] where similar results were found in which participants sought help from medical healthcare providers than non-Western healthcare providers such as traditional practitioners, despite the reports that 80% of South Africans consult traditional healers [29]. These two studies were from participants that had non-auditory chronic conditions in two different geographic locations. Investigations on adults with hearing impairment from other countries report similar findings to the current study [6, 8, 19]. However, Sorketti and colleagues [12] compared help-seeking in HICs and LMICs and reported traditional healers as the predominant first contact for participants in LMICs. Although comparing HICs and LMICs sheds light on the realities of different contexts in terms of help-seeking, this study [12] may have been too generalized in its comparison of LMICs. A further analysis per region may have provided a different outcome. It is important to highlight that the site of data collection (a Western healthcare site) most likely had an influence in the findings, and that had the site been an *indumba*, an African traditional healing clinic, different results may have been found – an important implication for future studies. The current authors believe that the very fact that participants in the current study were recruited in a Western healthcare setting arguably presupposes an inclination towards a biomedical system, and thus possibly a Western inclined epistemological positioning towards health.

In understanding illness from the socio-cultural context, only one (4,3%) participant consulted traditional practitioners for his ear and hearing complaints. The perception, recognition and interpretation of his ‘problem’ as viewed from his cultural perspective informed how he sought help [27, 50]. This finding is generally reported in groups of people as opposed to an individual however the current study sample is too small, and from a peri-urban context as opposed to a rural context where traditional practice is more prevalent, to make any conclusive inferences. Nevertheless, to support the current findings, van der Hoeven et al. [13] report that only a third of their chronic study population in the same province visited informal networks. In addition, most participants who were from both rural and urban communities, when seeking help for a non-auditory chronic condition, visited traditional practitioners primarily for social problems and not for ill health [13]. This is despite de Andrade and Ross [51] revealing that traditional practitioners are involved and are consulted in the treatment of a variety of ear and hearing difficulties. Furthermore, participants from their study present to traditional healers for mainly cultural reasons. This is consistent with the current study’s participant’s explanation of the reasons for seeking traditional medicine for help. The reason for this difference highlights the cultural variations that exist indicating that participants understand their illness from their own contextual reality, which needs to be considered when investigating patterns of presentation in help-seeking. Such considerations will facilitate planning and implementation of preventive audiology services that are responsive and responsible to the context.

With regards to research in hearing help-seeking, the pattern of presentation found in the current study is not reported. This is mainly due to the dearth in contextually relevant studies as Laplante-Levesque and his colleagues [6] acknowledged as the limitation in not investigating cultural differences in their paper. The finding of a limited number of participants seeking help according to their cultural practices may be as a result of an evolving socio-cultural environment, illness attribution and urbanization in the South African context [26, 28, 52], and the still strongly held colonial views and practices. In addition, participants may have attributed their ear and hearing difficulties to cultural reasons but were not willing to disclose this in a Western medical setting, a setting where the data collection for the study was conducted. This was a noted limitation of the current study. As the data analysis indicated, three participants who used formal networks considered their cultural practices and beliefs on their journey but nevertheless did not report this as the initial pattern of presentation. Participants were rather frustrated with the help-seeking journey mostly because of the medical costs required, hence they then considered alternative help, which may be more accessible and affordable. Some may have also not wanted to appear as neglecting their spiritual beliefs over dependence on Western medical healthcare. Regardless the reason, individual preferences dictate whether to seek help and if so, with which helper [10, 53]. This implies that in a diverse country, the influence of culture cannot be ignored in the lives of those presenting with ear and hearing difficulties when presenting to medical professionals as it provides insight into the journeys of those seeking help.

Another pattern that emerged from the context of this study is that a few participants (13%) presented to their family and friends. These social networks supported participants to resolve their hearing difficulties. Culturally, such a pattern in presenting to family and friends has been found in other communities [18, 54]. The dominant socio-cultural model of social relationships in Asian contexts made participants prefer to present themselves to the social support around them than to professional support when faced with ill-health [54]. This dimension of communal support described as collectivism by Zhao et al. [18] reflects the cultural beliefs and the subsequent attitude towards help-seeking. However, the limited number of participants may also mean that the society is very individualistic. Considering this, the reliance on social support can also be attributed to three factors, namely emotional support, participants’ lack of knowledge regarding where to seek help and a comparing and contrasting of symptoms to verify the need for help [8, 20]. Participants presented to their close networks because they did not know where or with whom to consult, as evidenced by the limited number of participants that presented to ear and hearing specialists. Some may have needed emotional support from their families as they struggled to acknowledge their hearing impairment [8, 20]. The complexity revealed in these findings indicates that there are various factors that influence decisions to seek help. An in-depth investigation is required to understand the cultural, social, and spiritual views of those with hearing difficulties.

The strength of this study is that it has shed light on the importance of investigating individual behaviors towards help-seeking from each social context. Despite a country rich in diverse languages and cultures, participants presented themselves according to their immediate context and social environment, highlighting the importance of having resources (awareness programs, ear and hearing clinics/facilities) easily available for community access. Participants also revealed the status of hearing healthcare in South Africa in terms of the limited access to audiologists as a first option to resolving their hearing complaints. Additionally, findings from this study around limited reporting of seeking help from traditional healers highlight the importance of ensuring that research questions are asked from different perspectives and at different epistemological sites. Thus, current findings contribute and add to the limited body of literature on understanding illness behavior particularly ear and hearing disorders in context.

Limitations identified in the current study’s design and methodology included a small sample size, with most participants being over the age of 60 years and coming from a Western biomedical healthcare facility. The researcher also interviewed participants who had already started the process of seeking help, which may have limited an understanding of this phenomenon from those who had yet to seek help. More interesting insights could have been gathered from a bigger sample size, in different settings, and various age groups, implications for future studies.

## Conclusion

Current findings have indicated that the pattern of presentation for adults with hearing impairment in South Africa is influenced by their beliefs of a Western bio-medical viewpoint to explain their illness. The majority of participants from this peri-urban community, who were mostly older adults, presented themselves to healthcare professionals at various levels of healthcare in the public and private healthcare sectors. However, as indicated ear and hearing care specialists were the least to be contacted in this context due to capacity versus demand challenges around human resource capacity in these professions. Furthermore, the influence of culture was deemed minimal in their help-seeking behavior patterns for hearing impairment. Seemingly, even in a diverse country where culture, language and ethnicity are connected, this did not impact their decisions. Though some participants expressed a belief in religious faith or traditional medicine, a pattern of valuing medical over traditional providers was observed in their responses. This implies that even in multi-cultural and multi-lingual contexts where evidence has indicated majority seeking primary healthcare from traditional healers, the help-seeking behavior patterns of individuals with hearing impairment cannot be presumed to follow the same pattern. Various reasons for this position, including the influence of colonialism, social stigma, access, and so on need careful consideration. The very fact that the study was conducted in a Western bio-medical healthcare setting is a significant variable to consider. Research on this important subject needs to reflect the dynamics of each geographic location as a combination of personal factors, contextual factors and social networks that together produce a pattern for seeking help [16, 55]. Audiologists need to consider these factors by being more responsive to their patients’ social context which will lead to improved interventions [55, 56], with preventive care being adopted within these interventions. Replication of the current study with the data collection site being the non-Western healthcare setting is important. While focusing on preventive healthcare, hearing health policy needs to be informed by the realities of help-seekers and the dynamics of each social context. Key to this is ensuring that awareness programs and ear and hearing facilities are brought closer to the communities so that they are contextually relevant and responsive to the realities of those communities. Future studies on this topic need to be located in a context that has a different epistemological positioning such as in deep rural areas of South Africa, and/or recruit participants from an *indumba* rather than a Western healthcare facility.

### Electronic supplementary material

Below is the link to the electronic supplementary material.

Supplementary Material 1

## Data Availability

All data generated or analyzed during this study are included in this published article.

## Abbreviations

HICs: High income countries
LMICs: Low-and-middle income countries
WHO: World Health Organization
ENT: Ear-Nose-Throat Specialist
PHC: Public Health Clinic
WMA: World Medical Association
HPCSA: Health Professions Council of South Africa
PTA: Pure Tone Average
