# Supplementary material for: Patterns of presentation of adults with hearing impairment in a peri-urban community in South Africa: a qualitative study
Source: BMC Health Serv Res. 2023 Sep 21;23:1019. doi: 10.1186/s12913-023-10025-5 (PMC10515004; doi:10.1186/s12913-023-10025-5)
Supplement: Supplementary file 1 — Supplementary Material 1 [file 12913_2023_10025_MOESM1_ESM.docx]

**Interview Guide**

**Title:** Patterns of presentation of adults with hearing impairment in a peri-urban community in South Africa: A qualitative study

**Biographical information**

Participant code: ______

Gender: Male or Female

DoB: ______________

Ethnicity: ______________

Degree of Hearing loss: ____________

Occupation: Unemployed/ Employed/Self-employed:

Student: Yes or No.

Participant: alone or accompanied by significant other

I would like you to tell me about your journey of seeking help for your hearing difficulties.

1. Tell me about the time when you started noticing that there was a problem you’re your ear/hearing.
2. Who did you first seek help from?

A: Doctor

B: Traditional Healer/Sangoma/Inyanga

C: Herbalist

D: Pastor/Imam/Priest

E: Ancestors

F: Family

G: Other

1. probe for pattern of presentation-first consulter.
2. What did they say/do?
3. From the response above, what was the reason for going to the ____? Some people will go to ______. How is this related to what you believe about how you treat any sickness in your body? What do you believe about what you do when you are sick?
4. Describe the journey that you took or followed from the very first sign/s that made you see that there was a problem (first symptom/s) to the audiologist/reaching this hospital?
5. Probe for pathway or process taken.
6. How did they actually seek help? How was the process (facilitators and barriers)?
7. What do you think were some of the barriers to you getting help for your hearing difficulties? Barriers to seeking help for your hearing difficulties. (what were the things that blocked you/prevented you/stopped you from getting help?)
8. Probe description in the journey, before reaching the audiologist.
9. Any barrier according to the patient including context specific barriers.
10. What do you think were some of the things that made it easier for you to get help for your hearing difficulties? Facilitators to seeking help for your hearing difficulties. (what were the things that helped you from getting the help?)
11. Probe description in the journey, before reaching the audiologist.
12. Any facilitator according to the patient including context specific barriers.
